# Treatments and uses of *Moringa oleifera* seeds in human nutrition: A review

**DOI:** 10.1002/fsn3.1057

**Published:** 2019-05-07

**Authors:** Romuald Willy Saa, Edith Nig Fombang, Elie Baudelaire Ndjantou, Nicolas Yanou Njintang

**Affiliations:** ^1^ Department of Food Sciences and Nutrition, National School of Agro‐industrial Sciences (ENSAI) University of Ngaoundere Ngaoundere Cameroon; ^2^ Department of Biological Sciences, Faculty of Science University of Ngaoundere Ngaoundere Cameroon

**Keywords:** food applications, moringa flour, moringa oil, *Moringa oleifera* seeds, technological treatments

## Abstract

This work reviews treatments and uses of *Moringa oleifera* seeds in human nutrition. *Moringa oleifera* seeds are considerable sources of proteins (mean 19%) and lipids (mean 31%). Previous reports presented the nutritional properties of the seeds and oil. Moringa seeds are sources of lipids, and their removal leads to Moringa seed flour with a high protein content which might play a role in food technology and human nutrition. Moringa oil has been tested in frying and was found to be more stable than groundnut oil; its incorporation in groundnut at level lower than 10% improved on the acceptability of chips. Several treatments like roasting, germination, and boiling have been applied to Moringa seeds to produce flour with improved nutritional properties. In particular, defatted Moringa flour has been applied in different formulations including cakes, cookies, burgers, infant porridges. Generally, the products deriving from the flour were more stable in conservation and well accepted for low substitution while high substitution increased the bitterness. Notwithstanding their high content in protein and oil, defatted *M. oleifera* seed flour and oil are still fairly investigated in order to envisage their integration in the food habits of people. The present wrote up reviews the treatments applied on *M. oleifera* seeds and applications of the defatted *M. oleifera* flour and oil in food systems for human nutrition.

## 1 INTRODUCTION

*Moringa oleifera* (Moringaceae) belongs to the genus Moringa which among the 13 species is the most widely used. *Moringa oleifera* is known worldwide under several popular names such as horseradish tree, drumstick tree, “Guiligandja,” “Gagawandalahai,” and many others (Morton, 1991). *Moringa oleifera* Lamarck or *Moringa pterygosperma* Gaertner is a South Asian plant native to the Himalaya Mountains, from Northwest Pakistan to North India (Ramachandran, Peter, & Gopalakrishnan, 1980). This plant is now cultivated in all tropical and subtropical regions such as Pakistan, Arabia, Central America, North and the South Philippines, Cambodia, Caribbean Islands, and Africa (Morton, 1991; Mughal, Ali, Srivastava, & Iqbal, 1999). This is due to its resistance to different climates, poor and averagely dry soils, and the multiple properties which abound to this plant (Morton, 1991; Mughal et al., 1999; Sengupta & Gupta, 1970). It grows and reaches 15 m in height, with a diameter of 20–40 cm at chest height. It produces dry fruits, triangular in shape, making seed dispersion by the wind easier (Odee, 1998). Many parts of the plant show pharmacological properties, recognized by popular use and corroborated by the scientific community.

Originally, this plant was cultivated for its leaves, whose nutritional potential is exploited to fight against malnutrition (Fuglie, 2002; Ndong, Wade, Dossou, & Guiro, 2007). In addition, leaf extracts show hypocholesterolemic and hypotensive activities (Gilani et al., 1994; Mehta, Balaraman, Amin, Bafna, & Gulati, 2003). The seeds show antimicrobial activity against fungi and bacteria (Donli & Dauda, 2003), and antitumor (Bharali, Tabassum, & Azad, 2003) and anti‐inflammatory activity. Studies on seeds are much accentuated on the purification of water and the oil extraction. The oil has a good stability for cooking and good technological aptitudes for frying (Abdulkarim, Long, Lai, Muhammad, & Ghazali, 2005; Anwar, Bhanger, & Kazi, 2003). However, studies on the nutritional composition and functional properties of defatted *M. oleifera* flour are scarce and show that they are a good underexploited protein source (Govardhan Singh, Ogunsina, & Radha, 2011).

This article reviews the treatment and application of *M. oleifera* seeds in human nutrition. The originality of this review compared to previous review is the emphasis on the food application of the defatted Moringa flour and oil. For our point of view, this is the first review on the application of defatted Moringa flour and oil in food systems. A recent review by Leone et al. (2016) reports the characteristics and uses of *M. oleifera* seeds and oil for human health, while previous review discussed the properties of the seeds without a link to seed treatment.

## 2 NUTRITIONAL POTENTIAL OF *Moringa* SEEDS

Beyond the interesting presence of proteins, lipids, and carbohydrates, *M. oleifera* seeds (Table 1) contain vitamins A and B1 (Mbah, Eme, & Ogbusu, 2012). They are also sources of minerals, micronutrients, and bioactive compounds such as flavonoids, saponins, sterols, phytates, and trypsin inhibitors. The seed could be considered as oilseeds from its lipid content varying from 13% to 46%. This presents *M. oleifera* seeds not only as a protein source but also as a source of lipids and fibers (Compaoré, Nikièma, Bassolé, Savadogo, Hounhouigan, et al., 2011; Compaoré, Nikièma, Bassolé, Savadogo, Mouecoucou, et al., 2011).

**Table 1:** Nutritional composition of whole *Moringa oleifera* seeds

Constituents	References				
	Compaoré, Nikièma, Bassolé, Savadogo, Hounhouigan, et al. (2011) and Compaoré, Nikièma, Bassolé, Savadogo, Mouecoucou, et al. (2011)	Mbah et al. (2012)	Abiodun et al. (2012)	Ijarotimi et al. (2013)	Bridgemohan et al. (2014)
Humidity (%)	2.1	6.8	4.7	10.6	5.4
Ash (%)	5.0	2.6	4.1	4.8	3.7
Fibers (%)	4.7	1.4	7.7	5.0	2.6
Lipids (%)	43.6	30.6	45.8	13.4	38.2
Proteins (%)	35.4	26.7	28.0	18.9	37.2
Carbohydrates (%)	9.2	32.0	10.6	53.4	12.9

### 2.1 Proteins

Proteins are the main source of nitrogen for human nutrition by bringing the essential amino acids for construction and renewal of body tissue (Biesalski & Grimm, 2010). *Moringa oleifera* seeds are a protein source, and they represent the second major component of these seeds after lipids. Recent studies reported protein content varying between 18.6% (Kawo et al., 2009) and 37.2% (Bridgemohan, Bridgemohan, & Mohamed, 2014). However, the analysis of defatted *M. oleifera* seed showed a protein content varying from 32% to 62.8% (Anwar & Rashid, 2007; Govardhan Singh et al., 2011). However, the protein composition of the seeds can cover only the requirements in some essential and semiessential amino acids for humans (histidine, threonine, tyrosine, leucine, isoleucine, phenylalanine) except methionine, lysine, valine, and tryptophan, considered as limiting amino acids (Table 2).

**Table 2:** Amino acid composition of *Moringa oleifera* seeds

Amino acids	*Moringa oleifera s*eed (Bridgemohan et al., 2014)	Dehulled and defatted (Bridgemohan et al., 2014)	*Moringa oleifera s*eed (Ijarotimi et al., 2013)	Fermented seed (Ijarotimi et al., 2013)	Spouting seed (Ijarotimi et al., 2013)	*Moringa oleifera* seed (Okereke & Akaninwor, 2013)	OMS/Food and Agriculture Organization (2001)
Nonessential amino acids							
Alanine	1.37	1.67	5.16	6.29	5.42	3.23	
Aspartate	1.46	1.78	15.70	21.37	18.13	6.14	
Serine	–	–	3.06	3.53	3.17	4.25	
Glutamate	6.84	8.53	17.87	22.46	20.23	14.76	
Proline	1.73	2.11	2.18	3.75	2.68	–	
Glycine	1.80	2.18	2.37	3.02	2.63	5.00	
Arginine	–	–	8.28	9.66	8.66	8.06	
Cysteine	1.52	1.84	1.68	2.02	1.79	2.02	
Tyrosine	–	–	1.97	2.34	2.09	2.33	
Histidine	–	–	1.93	2.94	2.50	2.01	2.1
Total	–	–	60.20	77.38	67.30	–	
Essential amino acids							
Lysine	0.55	0.64	0.31	0.41	0.36	3.24	4.2
Threonine	0.75	0.87	3.02	3.93	3.35	3.22	2.8
Valine	1.34	1.65	1.08	1.64	1.25	3.09	4.2
Methionine	0.70	0.84	0.31	0.41	0.35	0.97	2.2
Isoleucine	1.12	1.36	4.23	5.14	4.69	4.35	4.2
Leucine	1.93	2.36	3.83	5.04	4.08	5.27	4.2
Phenylalanine	–	–	3.27	4.25	3.57	4.53	2.8
Tryptophan	–	–	ND	ND	ND	–	
Total			16.05	20.82	17.65		

Treatments such as fermentation and germination increased all the amino acids (Table 2). The nutritional property of Moringa seeds can be improved through complementation with other foods rich in sulfur amino acids or lysine. In this respect, *M. oleifera* seed can be combined with cereals (rice, corn, sorghum, millet) to produce complementary foods with balanced proteins.

### 2.2 Carbohydrates

*Moringa oleifera* seed contains between 9.17% and 53.36% of carbohydrate (Compaoré, Nikièma, Bassolé, Savadogo, Hounhouigan, et al., 2011; Compaoré, Nikièma, Bassolé, Savadogo, Mouecoucou, et al., 2011; Ijarotimi, Adeoti, & Ariyo, 2013). The fibers which are nonavailable carbohydrates for the organism represent approximately 24% of dry weight of nondehulled seed and 3% of dehulled seed (Bridgemohan et al., 2014). The profile in mono‐ and disaccharides shows that *M. oleifera* seeds have low contents of glucose (2.57 g/100 g dw), fructose (0.03 g/100 g dw), and sucrose (2.91 g/100 g dw) compared to other medicinal plants such as pulps of *Adansonia digitata* (6.96, 4.03, 21.63 g/100 g dw) and *Parkia biglobosa* (13.55, 18.51, 24.07 g/100 g dw, respectively). Thus, *M. oleifera* seeds could be used in diabetic food (Compaoré, Nikièma, Bassolé, Savadogo, Hounhouigan, et al., 2011; Compaoré, Nikièma, Bassolé, Savadogo, Mouecoucou, et al.^2011^).

### 2.3 Vitamins and minerals

Studies by Mbah et al. (2012) showed that *M. oleifera* seeds contain provitamin A (2.04%) and vitamin B group, in particular B1 or thiamin (0.94%). Vitamin A plays a key role in vision and possesses antioxidant properties in the form of β‐carotene by limiting oxidation of molecules such as vitamin E. Vitamin E has been reported in *M. oleifera *seed oil in the forms of alpha‐tocopherol, gamma‐tocopherol, and delta‐tocopherol.

*Moringa oleifera* seeds are rich in minerals (Table 3) of which potassium, phosphorus, sodium, zinc, magnesium, and calcium are the principal minerals. Ijarotimi et al. (2013) revealed that *M. oleifera* seeds exhibited a Ca/P ratio higher than 1, while the Na/K ratio is higher than the recommended value (0.60) (Nieman, Butterworth, & Nieman, 1992). However, the mineral composition of *M. oleifera* seeds differs significantly (Table 3) from one region of the world to another.

**Table 3:** Mineral composition (mg/100 g) of *Moringa oleifera* seeds

Minerals	Kawo et al. (2009)	Compaoré, Nikièma, Bassolé, Savadogo, Hounhouigan, et al. (2011) and Compaoré, Nikièma, Bassolé, Savadogo, Mouecoucou, et al. (2011)	Abiodun et al. (2012)	Ijarotimi et al. (2013)
Calcium	602	78	20.38	128.33
Phosphorus	–	525	–	103.33
Iron	–	12.77	3.10	7.33
Sodium	86.2	25.01	15.50	295.10
Potassium	732	48.2	47.90	52.33
Magnesium	–	261	22.01	26.33
Copper	–	54.2	–	0.63
Zinc	–	300.47	0.81	0.11
Iodine	–	–	–	–
Manganese	17.5	95.40	0.3	–

### 2.4 Lipids

The level of lipids in *M. oleifera* seeds was reported between 14% and 46% dwb (Abiodun, Adegbite, & Omolola, 2012; Ijarotimi et al., 2013). The lipids are low in monounsaturated and saturated fatty acids but higher in polyunsaturated fatty acids representing up to 75%–79% (Table 4) (Ijarotimi et al., 2013). However, the oil is a source of some minor compounds (phytosterols and tocopherols). Several studies investigated the role of *M. oleifera* seed oil in human nutrition, and this includes physicochemical characteristics of the oil and its biological value (Abdulkarim et al., 2005; Andrade et al., 2011; Anwar & Rashid, 2007; Arafat, 2013; Compaoré, Nikièma, Bassolé, Savadogo, Hounhouigan, et al.^2011^; Compaoré, Nikièma, Bassolé, Savadogo, Mouecoucou, et al.^2011^).

**Table 4:** Acid fatty contents of *Moringa oleifera* seeds (expressed in percentage of total fatty acids)

Acides gras	References								
	Tsaknis, Lalas, Gergis, Dourtoglou, and Spilitois (1999)	Lalas and Tsaknis (2002)	Lalas and Tsaknis (2002)	Anwar and Bhanger (2003)	Abdulkarim et al. (2005)	Anwar and Rashid (2007)	Compaoré, Nikièma, Bassolé, Savadogo, Hounhouigan, et al. (2011) and Compaoré, Nikièma, Bassolé, Savadogo, Mouecoucou, et al. (2011)	Arafat (2013)	Olive oil
C8	0.03	–	0.03	–	–	–	0.04	–	–
C14:1c9	–	–		–		–	–	0.29	–
C14:0	0.11	–	0.13	–	0.1	–	0.1	0.30	<0.01 (<0.1)
C16:1c9	1.57	1.36	1.45	1.00	2.2	0.97	1.28	02.07	– (0.3–3.5)
C16:0	6.04	6.46	6.46	6.50	7.8	6.45	5.57	09.04	11.2 (7.5–20)
C17:0	0.09		0.08	–	–	–	0.1	–	0.1 (<0.5)
C18:2 c9,12	0.73	0.65	0.65	1.29	1.1	1.27	0.95	0.10	4.2 (3.5–21)
C18:3c9,12,15	0.22	0.18	0.18	–	0.2	0.30	0.45	32.82	0.5 (<1.5)
C18:1c9	73.6	71.21	71.21	76.00	67.9	73.22	72.4	42.43	72.21 (55–83)
C18:0	4.14	5.88	5.88	5.67	7.6	5.50	3.84	02.27	2.80 (0.5–5)
C20:0	2.76	3.62	3.6	3.00	4	4.08	3.4	01.61	0.6 (<0.8)
C20:1	2.4	2.22	2.22	1.20	1.5	1.68	2.7	–	0.2 (–)
C22:1	0.14	0.12	0.12	–	–	–	0.14	–	–
C22:0	6.73	6.41	6.41	5.00	6.2	6.16	6.95	02.89	<0.01 (<0.2)
C24:1	–	–	–	–	–	–	–	0.45	–
C24:0	1.08	–	–	–	1.3	–	1.5 8	–	– (<1)
C26:0	–	–	1.18	–		–	0.08	–	–
TAGS	20.95	22.37	23.77	20.17	27	22.19	21.66	16.11	–
TAGI	78.69	75.74	75.83	79.49	72.9	77.44	77.84	78.16	–
Total	99.64	98.11	99.6	99.66	99.6	99.63	99.5	94.27	–

Note

Fatty acids: C8: lauric; C14:c9: myristoleic; C14:0: myristic; C16:c9: palmitoleic; C16:0: palmitic; C17:0: heptadecanoic or margaric; C18:2 c9,12: linoleic; C18:3c9,12,15: α‐linoleic; C18:c9: oleic; C18:0: stearic; C20:0: arachidonic; C20:1: gadoleic; C22:1: erucic; C22:0: behenic; C24:1: nervonic; C24:0: lignoceric; C26:0: hexadodecanoic; TAGS: total of saturated fatty acids; TAGI: total of unsaturated fatty acids.

### 2.5 Biological activity of *Moringa oleifera* seed

Very few studies are reported on *M. oleifera* seed as proteins sources. Oliveira, Silveira, Vasconcelos, Cavada, and Moreira (1999) reported that consumption of crude seeds of *M. oleifera* induced deleterious effects in Wistar rats. They associated the toxicity of the seeds to lectin (hemagglutinin) previously reported in *M. oleifera* seeds (Santos, Argolo, Coelho, & Paiva, 2005). Ben Salem and Makkar (2009) observed that defatted Moringa seed, incorporated in meal at levels up to 4 g/day, had positive effects on rumen fermentation, digestion and performance of lambs. The authors equally observed a reduction in the overall performance of the lambs at a feeding level of only 6 g/day, and this was associated with the presence of glucosinolates. Besides, Igwilo et al. (2013) showed that 30 min soaked *M. oleifera* seeds did not support growth, and induced threat for the liver of Wistar albinos rats fed for 21 days. No study reported the utilization of Moringa seeds.

## 3 FUNCTIONAL PROPERTIES OF *Moringa oleifera* SEEDS

Ijarotimi et al. (2013) evaluated the effect of germination and fermentation on the functional properties of *M. oleifera* flour. The apparent mass density 0.45 g/ml (measured without packing) and the true mass density of *M. oleifera* seeds (0.63 g/ml) did not vary significantly with seed fermentation and germination (Ijarotimi et al., 2013). As some leguminous seeds (bambara groundnut: 0.60–0.75 g/ml), the flour of *M. oleifera* seeds has a low mass density (Onimawo & Egbekun, 1998).

The foaming capacity of *M. oleifera* seed flour significantly increase with fermentation (25.93%–29.63%) and germination (25.93%–37.70%) (Ijarotimi et al., 2013). Ijarotimi et al. (2013) equally reported increased swelling capacity following fermentation (from 1.27 to 1.50), while germination had no significant effect on it (1.27%–1.33%). The water absorption capacity (WAC) of *M. oleifera* seed flour was 80.3 g/ml lower than that of most cereal and legume flours. Fermentation increased the WAC to 141 g/ml, while germination had no significant effect (Ijarotimi et al., 2013). Besides, Ogunsina, Radha, and Sign (2010) compared the functional properties of full‐fat and defatted *M. oleifera* seed flour. The nitrogen solubility was lowest, that is, 27.8% and 29.2% at pH of 4.0 and 9.0 respectively for full‐fat and defatted *M. oleifera* seed flour, while maximum solubility occurred at pH 6.0. Defatting increased the water absorption (115.7–130.5 g H_2_O/100 g) and fat absorption (129.8–208 g oil/100 g) capacities of *M. oleifera* seed flour. The foaming capacity and foam stability of the defatted flour were 86.0% and 82.0 ml, whereas those of full‐fat flour were 20.6% and 18.5 ml, respectively. The defatted flour showed better emulsification (97.2 ml/g) than full‐fat flour (66.0 ml/g). The least gelation concentrations of the defatted and full‐fat flours were 14% and 16% (w/ v), respectively.

Oloyede, James, Ocheme, Chinma, and Akpa (2015) showed that 71‐hr natural fermentation of defatted *M. oleifera* seed flour increased significantly the WAC (0.86–2.31 g/ml), oil absorption capacity (0.87–1.91 g/ml), foaming capacity (9.76%–16.31%), and emulsifying capacity (50.71%–68.75%). Concomitantly, natural fermentation induced a significant decrease in bulk density (0.53–0.32 g/cm^3^) and dispersibility (36.00%–20.50%). Significant increases in pasting properties of the flour with fermentation were also reported: peak viscosity (15–34 RVU), true viscosity (11–21.5 RVU), breakdown viscosity (4–14.5 RVU), final viscosity (16–36 RVU), and setback (5–17.5 RVU).

Globally, studies on the functional properties revealed that defatted Moringa flour exhibited higher foaming capacity and stability, higher emulsifying capacity, and lower WAC and viscosity. In addition, the fat absorption capacity was significantly higher than the WAC, making Moringa flour a potential stabilizer for food emulsion and foaming systems.

## 4 Comprehensive Food ScienceTREATMENTS AND CHEMICAL COMPOSITION OF *Moringa oleifera* Seed

The *M. oleifera* seeds have a bitter taste and contain antinutritional factors which reduce digestibility making raw *M. oleifera* seeds/flour unsuitable for consumption. Some treatments were reported on Moringa seeds to reduce their antinutriments and bitterness which can make them easy to use as food or food ingredients (Ijarotimi et al., 2013), the goal that we want to achieve with the improvement of the nutritional quality of the seeds and oil.

### 4.1 Heat treatments (cooking and roasting)

#### 4.1.1 Cooking

Mbah et al. (2012) studied the composition of *M. oleifera* as affected by cooking time (10, 20 and 30 min), and they observed 20% reduction of the lipids and ashes due to the leaching during cooking, and an increase in proteins from 26.7% to 32.0%. Heating *M. oleifera* seeds at temperatures 100, 130, and 150°C during 30 min induced an increase in oil content from 28.9% to 33.7%, 32.2%, and 30.9%, respectively (Adejumo, Alakowe, & Obi, 2013). In addition, the physicochemical parameters of oil were also significantly affected. In particular, oil density diminished from 1.05 to 0.99, 0.97, and 0.95 kg/m^3^; the saponification index diminished from 252.3 to 230.8, 218.8, and 177.7; the free fatty acids diminished from 5.80 to 2.74, 2.70, and 2.71 mg/KOH/g; the acid value diminished from 2.73 to 1.37, 1.36, and 1.35 mmol/kg; and the iodine index changed from 72.4 to 69.3, 66.7, and 66.6. In conclusion, heating had no significant influence on the specific gravity and density of *M. oleifera* seeds oil while it reduced the physicochemical characteristics (Adejumo et al., 2013).

#### 4.1.2 Roasting

Mbah et al. (2012) showed that roasting of *M. oleifera* seeds increased the calcium, zinc, iron, lipids, carbohydrate, fiber, and ash contents, the percentage change being higher as the roasting time increased. The increase was assigned to the loss of water during roasting. Besides, vitamin A and B1 levels significantly decreased, while an increase in protein content was observed after 20 min of roasting. Significant reduction in tannins and increase in saponin and phytate contents were observed after roasting. On the other hand, the oxalate content increased initially (3.58% at 10 min) before dropping gradually to reach the value of raw seeds (2.87% at 30 min and raw seeds). In addition, this treatment (roasting at 70°C during 15 min) was also used by Compaoré, Nikièma, Bassolé, Savadogo, Hounhouigan, et al. 2011 and Compaoré, Nikièma, Bassolé, Savadogo, Mouecoucou, et al. 2011 on *M. oleifera* seeds in order to eliminate their bitter taste for infant flour formulation and porridge preparation.

### 4.2 Biological treatments

#### 4.2.1 Fermentation

Ijarotimi et al. (2013) studied the effect of natural fermentation on the proximate composition of *M. oleifera* seeds. The protein content increased from 18.9% to 21.2%. Similarly, the carbohydrate content increased from 53% to 61%, while no significant change was observed on the lipid content (13.4%–14.0%), the crude fiber content (5.0%–6.2%), and ash content (4.8%–4.5%). In accordance with the decrease in ash content, significant reductions of almost all the minerals (mg/100 g) of seed were observed (Ijarotimi et al., 2013): calcium (128.33–121.67), phosphorus (103.33–91.67), iron (7.33–5.63), sodium (295.1–280.3), potassium (52.33–43.67), magnesium (26.33–25.13), copper (0.63–0.57), and iodine (0.11–0.10). This also affected some mineral ratios such as sodium/potassium ratio (5.65 to 6.42) and calcium/phosphorus (1.24–1.33) which are indicators of body balance and bone formations (Nieman et al., 1992). Irrespective of the treatment, the Na/K ratios were higher than 1, suggesting *M. oleifera* seeds should be prohibited in people with risk of hypertension (Ijarotimi et al., 2013). The Ca/P ratio higher than 1, along with the contents in Ca and P (Ijarotimi et al., 2013), gives *M. oleifera* seeds advantage to support growth of children (Nieman et al., 1992).

The protein content in *M. oleifera* seeds increased during fermentation, but the amino acids lysine and methionine were in all cases the limiting amino acids (Table 2). In addition, the fatty acid profile of *M. oleifera* seeds significantly varied with fermentation, the polyunsaturated fatty acid increasing from 58.8% to 62.1%, the saturated from 26.8% to 28.7%, and the monounsaturated from 13.54% to 8.54% (Ijarotimi et al., 2013).

Most of the secondary metabolites equally diminished during fermentation of *M. oleifera* seeds. Thus, phytates (78.33%–28.33%), tannins (241.67%–146.67%), polyphenols (40%–23%), alkaloids (17.33%–12.33%), flavonoids (5.50%–5%), and saponins (9.83%–7.50%) drop under the effect of fermentation except terpenoids (20%–27.50%) (Ijarotimi et al., 2013). The low palatability of *M. oleifera* seeds was attributed to its high tannin content in which the decrease during fermentation might improve its acceptability when incorporated into food (Mehansho, Buttler, & Carbon, 1987). Comparatively, alkaloid content of *M. oleifera* seeds was lower compared to recommended level of 60 mg/100 g for healthy food (McDonald, Edwards, Greenhalgh, & Morgan, 1995).

#### 4.2.2 Germination

Germination is a normal biological process of plants by which the seeds leave the latency stage (Sangronis & Machado, 2007). During germination, some changes occur in terms of quantity and type of nutrients in seed. These changes could be due to the type and variety of seed and germination conditions (Dhaliwal & Aggarwal, 1999). An increase of minerals, increase of protein bioavailability, and a reduction of secondary metabolites of foodstuffs are observed during germination (Hassan, Babiker, & Tinay, 2007; Kouakou, Alexis, Adjehi, Marcelin, & Dago, 2008). Several studies reported the effect of germination on the nutritional properties of *M. oleifera* seeds. Germination had no significant effect on ash (4.8%–4.3%), fiber (5.0%–5.5%), and carbohydrate (53.4%–53.0%) contents. However, the protein (18.9%–23.7%) and lipid (13.4%–14.6%) contents significantly increased with germination (Ijarotimi et al., 2013). Significant reduction in the tannin (247.6%–181.7%), phytate (78.33%–40%), polyphenol (40%–34.3%), alkaloid (17.3%–15.3%), and saponin (9.8%–8%) contents was equally reported (Ijarotimi et al., 2013). According to Chinma, Gbadamosi, Ogunsina, Oloyede, and Salami, (2014), germination of 12h with changing of water all the 2 hr in order to avoid any spontaneous fermentation reduced the bitterness and astringency caused by antinutrients.

## 5 UTILIZATION OF *Moringa oleifera* SEED FLOUR AND OIL IN FOOD SYSTEM

### 5.1 Oil

Moringa seed oil gathered good thermal, oxidative, and frying stabilities. Ogunsina et al. (2014) studied the effect of extraction method and temperature, and storage time on the physicochemical properties of Moringa oil. The peroxide index of *M. oleifera* oil Var *Jaffina* during 42 days of storage varied from 1.2 to 5.6 meq O_2_/kg. This variation was significantly lower than that of groundnut oil which was 3–26.93 meq O_2_/kg in the same condition of extraction and storage. The higher stability of Moringa oil when compared to groundnut oil may be attributed to their lower polyunsaturated fatty acid level. Although unsaturation in fatty acid generally increases the susceptibility of oil to oxidation, polyunsaturated fatty acids are the most incriminated in the oxidative stability of oil (Bhatnagar, Prasanthkumar, Hemavathy, & Gopalakrishna, 2009). Abdulkarim , Long, Lai, Muhammad, and Ghazali (2007) assay the use of Moringa oil in comparison with groundnut oil in potato frying. They observed that the free fatty acids increased for 28.6% in Moringa oil and 48.6% in groundnut oil. The increase in free fatty acid during frying is a normal reaction which starts with water liberation from the product being fried followed by thermal hydrolysis of the acylglyceride into fatty acid and glyceride. The mechanism involves nucleophilic attack of the ester bond of acylglycerol by water (Choe & Min, 2007). Whatever the case, Moringa oil is less likely to hydrolysis than groundnut oil. In this respect, cold‐pressed oils from *M. oleifera* seeds are better than the raw commercial oils and refined groundnut oils. In the same vein, Khattab and Shakak (2012) showed that during frying of potato chips, *M. oleifera* oil was more stable to oxidation (based on peroxide, free fatty acid, density, viscosity, and refraction index) compared to groundnut oil and the mixture (1/1 ratio) of both. In addition, the acceptability (taste, color, odor, texture, and general acceptability) of potato chips analyzed by a panel of seventeen students was higher as compared to those made from groundnut oil, but lower than that obtained from the mixture of Moringa and groundnut oils (Khattab and Shakak, (2012). Based on the current results, *M. oleifera* seed oil showed enough promises to be regarded as a more stable and healthy oil in cooking and frying.

### 5.2 Flour

Chinma et al. (2014) studied the addition of germinated *M. oleifera* seed flour to wheat flour for cake production. Increase in Moringa level up to 40% increased the protein content from 13.14% to 23.10%. In addition, the fiber, iron, zinc, calcium, lipid, and ash contents also increased significantly with increase in Moringa level in the blend. In contrast, the increase in the level of *M. oleifera* reduced gradually the pasting properties in general, and particularly peak, final, and breakdown viscosities. The cake made from the blend showed significant decrease in acceptability. The bitter and astringent tastes were more pronounced on 40% substitution, while up to 30% substitution, the cake were rated favorably (Chinma et al., 2014).

Ogunsina, Radha, and Indrani (2011) evaluated the effect of wheat flour replacement with debittered *M. oleifera* flour for cookies and bread formulation. *Moringa oleifera* seeds were debittered by cooking at 100°C for 35 min. Breads were more accepted at mixture wheat/Moringa ratio 90/10, while cookies were accepted at ratio 80/20. The bread had an acceptable characteristic taste of *M. oleifera* seed, while cookies had a hazelnut after‐taste in the mouth. The products were rich in protein and other essential nutrients such as iron and calcium which are seldom found in daily diets. Thus, the substitution rate of *M. oleifera* seed flour depends on the treatment applied with *M. oleifera* seed germination favoring a high substitution rate compared to seeds boiling (Ogunsina et al., 2011).

Al‐Juhaimi, Ghafoor, Hawashin, Alsawmahi, and Babiker (2015) studied the addition of *M. oleifera* seed flour (2%, 4%, and 6%) in beef burger preparation and found no significant change on the sensory attributes (appearance, juiciness, flavor, taste, tenderness, and overall acceptability). Likewise, addition of *M. oleifera* flour in burger increased the shelf life. In this respect, while burger with 0% and 2% Moringa had respectively 7 and 17 days of conservation, burger with 4% and 6% had 21 days of conservation. The increased conservation of burger with Moringa flour was attributed to the presence of antioxidants from Moringa flour (Al‐Juhaimi et al., 2015). In this respect, Moringa flour may play a role as ingredient for food conservation.

Roasted *M. oleifera* seed flour was equally tested in infant food formulation. According to Compaoré, Nikièma, Bassolé, Savadogo, Hounhouigan, et al. 2011 and Compaoré, Nikièma, Bassolé, Savadogo, Mouecoucou, et al. 2011, roasting at 70°C for 15 min reduced the bitterness. The formulations include mixture with (a) *Zea mays, Cucurbita maxima, M. oleifera,* pulps *of P. biglobosa,* pulps *of A. digitata* and sugar; (b) *Oriza sativa* L*., C. maxima, M. oleifera, pulps of P. biglobosa*, pulps *of A. digitata* and sugar; (c) *Pennisetum glaucum, C. maxima, M. oleifera,* pulps *of P. biglobosa,* pulps *of A. digitata* and sugar; and (d) *Misola*, *P. glaucum, Soja hispida, Arachis hypogea,* and sugar.

Jude‐Ojei, Lola, Ajayi, and Seun (2017)studied supplementation (10%, 20%, and 30%) of fermented (2 days of fermentation in ambient temperature) Moringa seed flour in the preparation of maize “Ogi,” which is a traditional fermented food from western Nigeria. They found that supplementation increased nutritional potential (protein, ash, and lipid) but reduced functional and pasting properties and moisture content of product, leading to increased shelf life. This favors the use of Moringa flour as a protein source to supplement local/traditional cereals such as maize, sorghum, millet (Jude‐Ojei et al., 2017). Similarly, Aluko, Brai, and Adelore (2013) evaluated the sensory attributes of the incorporation (2%, 5%, and 7.5%) of Moringa seed flour in the preparation of the maize snack (maize/Moringa snack). They found that increasing the Moringa rate increased the nutrient composition (protein, ash, lipid, fiber) and reduced some functional properties (swelling capacity, bulk density). The general acceptability of the snacks of the four different blends showed that snacks containing 7.5% Moringa flour were very well accepted by panelists in terms of color, crispness, taste, aroma, and general acceptability.

## 6 CONCLUSION AND FUTURE RESEARCH

*Moringa oleifera* seeds have been the subject of many research activities. Moringa seeds are sources of proteins, lipids, fats, soluble vitamins, and antioxidants. The proteins are particularly poor in lysine, sulfur amino acids, and tryptophan. The principal component of the oil is oleic acid, and the oil is perfectly tested in frying and seasoning. Based on their level in antioxidant in general, and in particular tocopherol, phytosterol, and carotenoids, the whole *M. oleifera* flour (2%–6%) also demonstrates preservative role in meat products. The seed is bitter and astringent; the untreated and soaked seeds are equally toxic when consumed. A process for debittering the seeds completely and used in some food preparation has been documented. Some research addressed the functional properties of *M. oleifera* seed flour with some technological applications in biscuits and infant flour formulations. However, the bitterness and toxicity of the flour are still a limit for their utilization. Furthermore, the oil is not only free of toxicant, but it also exhibited high biological value as compared to commercial oil.

With the global shortage of food grains and ever increasing human population, *M. oleifera* will certainly offer good alternative to food shortage. In particular, it easily grows under dry climates of the sub‐Saharan areas which generally future food crisis. For *M. oleifera* seeds to play that role, more research in the effect of treatments on the functionality and the nutritional and physicochemical properties of the oil and defatted seed flour should be addressed. For instance, the combined effect of roasting, germination, and cooking on the in vivo nutritional quality and toxicity of the flour and fat could be tested. Formulation of value‐added products from *M. oleifera* oils and flour, such as ingredients for preservation, nutraceutical with immunomodulatory and anti‐inflammatory actions, may also offer good perspectives to the seeds. Investigations of the effect of consumption of Moringa seeds and products enriched with Moringa seeds on some markers of toxicity in humans are also envisaged.

## CONFLICT OF INTEREST

The authors declare no conflict of interest.

## AUTHOR CONTRIBUTION

NYN, EBN, and RWS have made substantial contributions to conception and design, while RWS and ENF contributed to drafting the manuscript. All the authors critically revised and approved the final submitted version of the manuscript. Prior to submitting the article, all authors agreed on the order in which their names are listed in the manuscript.

## ETHICAL STATEMENT

This study does not involve any human nor animal testing.
